# Long-term (up to 18 years) effects on GH/IGF-1 hypersecretion and tumour size of primary somatostatin analogue (SSTa) therapy in patients with GH-secreting pituitary adenoma responsive to SSTa

**DOI:** 10.1111/j.1365-2265.2007.02878.x

**Published:** 2007-08-01

**Authors:** Jean Christophe Maiza, Delphine Vezzosi, Maria Matta, Florence Donadille, Florence Loubes-Lacroix, Maxime Cournot, Antoine Bennet, Philippe Caron

**Affiliations:** * Department of Endocrinology, CHU Rangueil Toulouse, France; † Department of Neuroradiology, CHU Rangueil Toulouse, France; ‡ Department of Epidemiology, CHU Toulouse Toulouse, France

## Abstract

**Context:**

The role of somatostatin analogues (SSTa) in the treatment of acromegaly.

**Objective:**

To evaluate the antihormonal and antitumour efficacy of long-term (up to 18 years) primary treatment with SSTa in patients with GH-secreting pituitary adenoma responsive to SSTa.

**Design:**

An open, prospective, single-centre, clinical study.

**Patients:**

Thirty-six acromegalic patients, aged 17–75 years (postoral glucose tolerance test GH > 1 µg/l, increased IGF-1 for age and sex), were monitored in a single centre and treated with SSTa as first-line therapy. The mean pretreatment GH level was 13·5 ±3·1 µg/l, and IGF-1 (as a percentage of the value over the normal range) was 302 ± 26%. The patients had macroadenoma (*n* = 25), microadenoma (*n* = 8) or empty sella turcica (*n* = 3). The mean duration of treatment was 8 years (range 3–18 years). Hormonal and morphological monitoring was undertaken after 6 months, and then the patients were followed annually.

**Results:**

After 1 year, the mean GH and IGF-1 levels had reduced considerably (GH: 2·4 ± 0·3 µg/l; IGF-1; 174 ± 14%, *P* < 0·01), and they continued to decrease over 10 years, with a mean GH level of 1·6 ± 0·1 µg/l and IGF-1 of 123 ± 18% (*P* = 0·02). GH < 2 µg/l, normal IGF-1, or both were observed in 25 (70%), 24 (67%) and 21 (58%) patients, respectively. The mean reduction in tumour volume was 43% (range 13–97%) and shrinkage > 20% was obtained in 21 patients (72%). SSTa treatment was well tolerated with few digestive or metabolic side-effects.

**Conclusion:**

Long-term (up to 18 years) treatment with SSTa used as first-line therapy is effective from both an antihormonal and antitumour perspective, and is well tolerated in acromegalic patients.

## Introduction

Acromegaly is a chronic disease due to a hypersecretion of GH and IGF-1, associated with an increase in mortality and morbidity, mainly through cardiovascular, metabolic and neoplasic complications.^1^ The increased morbidity and mortality is directly related to the control of the disease.^2^ The aim of treatment is to restore physiological secretion of GH and IGF-1 or at least limit the GH/IGF-1 secretion excess, in order to reduce morbidity or mortality.

According to most guidelines, a neurosurgical approach is considered as the first-line treatment of acromegalic patients because it is able to cure the acromegaly quickly and definitively.^3^,^4^ The results vary according to the volume of the adenoma, the initial levels of GH and the neurosurgeon's experience.^5^ In cases of GH-secreting microadenoma, the cure rate is 80% in the best series; in cases of macroadenoma a cure rate of 50% is achieved, but the rate is much lower if the adenoma is invasive.^6^ A second-line treatment is then necessary.

Conventional^7^ or stereotaxic radiotherapy^8^,^9^ is sometimes used in the treatment of acromegaly, but its efficacy is delayed and the side-effects can limit its use.

Medical treatment of acromegaly, frequently indicated as second-line therapy, is based mainly on somatostatin analogues (SSTa), for which the development of slow-release forms has simplified their use and improved compliance.^10^–^12^ SSTa are discussed as primary treatment when surgery is contraindicated or refused by the patient, and in patients with normal magnetic resonance imaging (MRI). The medical treatment also includes dopamine agonists with more modest effects on GH secretion,^13^ and more recently a GH receptor antagonist (pegvisomant) was developed, although its place in the therapeutic armamentarium still remains to be defined more precisely.^14^

The aim of the present clinical study was to evaluate the long-term antihormonal and antitumour efficacy as well as the safety of SSTa in a cohort of acromegalic patients responsive to SSTa and primarily treated with SSTa for a mean duration of 8 years (range 3–18 years).

## Materials and method

### Patients

Among the patients monitored in our centre from 1986 to 2003 (*n* = 106) for the treatment of acromegaly, only patients treated with SSTa as primary treatment for at least 3 years were included in this clinical study. Sixty-nine patients treated by surgery or radiotherapy were excluded: 39 patients had primary surgery and 22 had secondary surgery when GH/IGF-1 hypersecretion was uncontrolled by SSTa (*n* = 6), dopamine agonists (*n* = 7) or their combination (*n* = 9). Pituitary radiotherapy was performed in primary (*n* = 1) or after pituitary surgery (*n* = 26). One acromegalic patient had normal GH/IGF-1 function after an acute episode of pituitary apoplexy.

The cohort included 17 men and 19 women with a mean age of 53 ± 2·5 years (range 17–75) (Table 1). The biological diagnosis of acromegaly was based on a plasma GH concentration > 1 µg/l after oral administration of 75 g glucose, in association with a raised IGF-1 concentration for age and sex. Primary medical therapy with SSTa was initiated because of systemic complications that contraindicated surgery (*n* = 7), refusal by the patient to undergo surgery (*n* = 8), empty sella turcica (*n* = 3) or invasive pituitary macroadenoma after radiological evaluation (*n* = 18). The initial pituitary imaging revealed a macroadenoma in 25 patients, a microadenoma in eight patients and an empty sella turcica in three patients. Six patients had hyperprolactinaemia (> 20 µg/l) at the time of the diagnosis. The mean follow-up was 8 years (range 3–18).

**Table 1:** Patient characteristics, duration of treatment, GH and IGF-1 levels at diagnosis and at the last evaluation during long-term somatostatin analogue treatment

Patient	Sex	Age at diagnosis	Duration of treatment (years)	Status of adenoma	Initial GH (ng/ml)	Final GH (ng/ml)	Initial IGF-1 (% ULN)	Final IGF-1 (% ULN)
1	F	61	12	Macro	6	0·5	284	97
2	M	61	8	Macro	6·8	0·2	286	95
3	M	51	8	Micro	6·3	0·3	341	96
4	M	56	8	Macro	2·5	0·8	445	274
5	F	54	13	Macro	7	0·5	128	82
6	F	52	5	Macro	2·5	0·8	247	247
7	M	68	3	Macro	82	9·3	749	301
8	F	38	3	Macro	2·5	4	203	206
9	F	57	4	ES	8	0·8	167	95
10	F	45	3	Macro	35	10·6	234	217
11	M	17	10	Macro	6	0·3	116	40
12	F	63	5	ES	8	0·6	263	82
13	F	68	5	Macro	20	1	424	90
14	F	41	3	Macro	75	10	278	288
15	F	45	12	Micro	3·3	0·3	197	80
16	F	65	13	Macro	8·2	1·7	–	46
17	F	75	4	Macro	4·5	3·7	417	99
18	F	71	6	Macro	5·5	2	247	100
19	M	43	5	Macro	6·5	0·7	249	42
20	M	45	18	Micro	–	2	614	161
21	M	67	9	Macro	15	2	–	59
22	M	66	12	Macro	10	2·7	–	192
23	F	21	17	Macro	28	0·3	–	54
24	F	63	10	Micro	2·5	0·3	287	83
25	M	70	3	Macro	3·6	2·7	191	193
26	F	52	8	ES	2	0·3	353	100
27	M	28	10	Macro	16·2	2·1	247	153
28	M	28	3	Micro	14·5	3·1	–	84
29	M	57	4	Macro	14	2·2	307	200
30	F	74	3	Micro	9	3·8	56	57
31	M	42	16	Micro	6·2	1	–	91
32	M	49	7	Macro	4·4	1·8	358	163
33	F	62	12	Macro	5·3	1·8	341	100
34	M	41	4	Macro	40	0·8	526	98
35	F	70	3	Macro	2·9	1·4	254	88
36	M	62	11	Micro	3·5	1	262	92
Mean ± SEM		53 ± 2·5	7·8 ± 0·7		13·5 ± 3·1	2·7 ± 0·7	302 ± 26	126 ± 12

M, male; F, female; Macro, macroadenoma; Micro, microadenoma; ES, empty sella turcica.

The patients were assessed clinically for efficacy and safety, biologically and radiologically at the time of the diagnosis, 6 months after starting SSTa therapy and subsequently annually.

### Therapeutic protocol

In accordance with the protocol of the study, all patients were treated with SSTa initially and throughout the duration of the study. The treatments evolved over time and were as follows:

- Nineteen patients were initially treated with octreotide subcutaneously with a dosage between 300 and 1500 µg/day (Sandostatin®, Novartis Pharma, Rueil-Malmaison, France), 14 were switched to lanreotide Autogel, 60 and 120 mg/28 days (Somatuline Autogel®, Beaufour Ipsen, Paris, France) and one to octreotide LAR, 30 mg/28 days (Sandostatin LAR® Novartis Pharma) to improve compliance to treatment and quality of life.
- Twelve patients were initially treated with lanreotide Autogel, 60 mg/28 days, and the treatment was changed to octreotide LAR, between 20 and 30 mg/28 days, in three patients because of transient gastrointestinal symptoms or induration at the injection site.
- Five patients were initially treated with octreotide LAR, 20 mg/28 days, and the treatment was changed to lanreotide Autogel, between 90 and 120 mg/28 days, in three patients because of technical problems with the injections.

The dosage of SSTa was adapted according to the hormonal response, and for the slow-release formulations reassessed after the fourth injection. In the case of insufficient response (GH > 2 µg/l and/or IGF-1 not normalized), treatment was increased to the maximal dose (30 mg/28 days for octreotide LAR, 120 mg/28 days for lanreotide Autogel).

In addition, some patients received treatment with dopamine agonists during the study. In the six patients with hyperprolactinaemia, treatment with dopamine agonists was initiated at diagnosis and was as follows: bromocriptine 5–15 mg/day in two patients (Parlodel®, Novartis Pharma); quinagolide 75–150 µg/day in three patients (Norprolac®, Ferring, Gentilly, France); and cabergoline 1 mg/week in one patient (Dostinex®, Pfizer, Paris, France). In nine other patients, dopamine agonist treatment was initiated later to improve GH/IGF-1 control and distributed as follows: bromocriptine 5 mg/day in one patient, quinagolide 75–150 µg/day in seven patients, and cabergoline 3·5 mg/week in one patient.

### Hormonal data

For the patients treated with slow-release formulations of SSTa (Somatuline Autogel, Octreotide LAR), the hormonal assessment was carried out on the day before the injection.

At each visit, the serum GH level was calculated as the average of six successive GH samples, taken at 30-min intervals between 0900 and 1200 h. Before 2003, GH assays were performed using a radioimmunometric technique (Cisbio international, Schering, Bagnols sur Cèze, France). The detection limit was 0·02 µg/l; the intra- and interassay coefficients of variation (CVs) were 2·1% and 4·5%, respectively. From 2003, GH assays have been performed using a chemiluminescence technique (Nichols Institute Diagnostics, San Clemente, CA). The detection limit is 0·02 µg/l; the intra- and interassay CVs are 3·7% and 6·2%, respectively. With this assay, 3 mU/l corresponds to 1 µg/l. The rate of correlation between these two methods is as follows: (*r*) = 0·9269 with a regression formula *y* = 0·9127*x* – 0·4601, obtained from a linear regression analysis of the data.

At each visit, the IGF-1 level was assessed from a serum sample. Before 2003, the IGF-1 assay used a radioimmunometric technique (Diagnostic System Laboratories, Webster, TX). The detection limit was 0·8 µg/l; the intra- and interassay CVs were 1·5% and 3·7%, respectively. From 2003, the IGF-1 assay used a chemiluminescence technique (Nichols Institute Diagnostics). The detection limit is 6 µg/l; the intra- and interassay CVs are 5·2% and 5·7%, respectively. The rate of correlation between these two methods is as follows: (*r*) = 0·8819 with a regression formula *y* = 0·882*x* – 19·84, obtained from a linear regression analysis of the data.

The serum levels of PRL, TSH, T4, LH, FSH and testosterone were measured using commercial methods.

### Radiological data

The patients were evaluated at the time of the diagnosis, after 6 months of medical treatment and every year during the long-term follow-up. Most of the neuroradiological data were obtained by MRI. Some data – older or in the case of contraindication to MRI – were obtained by computed tomography (CT) scan. All the images were viewed by the same neuroradiologist (F.L.-L.), who was unaware of the therapeutic protocol followed by the patient. Adenoma volume was assessed by the Di Chiro formula:^15^ volume = π/6 × (transverse × anteroposterior × ventral). The MRI shots (Siemens Magneton; 1 T, Erlanger, Germany) were carried out in the coronal and sagittal planes of 3 mm thickness, T1 weighed SE before and after injection of gadolinium. The scanographic shots (Siemens Somaton Plus 4) were carried out in the coronal and sagittal planes, millimetrically.

The radiological data were analysable in 29 of the 36 patients; an empty sella turcica was evident in three patients and initial radiological data were not available in four patients.

A gall bladder ultrasound was carried out upon diagnosis, after 6 months, and then annually.

### Statistical data

Means are given with their standard error (SE). In the event of non-normal distribution of the continuous variables, the median and the interquartile range are presented. Comparisons of percentages were made using the Pearson χ^2^-test. Means were compared using the Student or Mann–Whitney tests (case of 2 means) and an analysis of variance or the Kruskal–Wallis test (case of more than 2 means). Nonparametric tests were used when the variables were not distributed normally or in the event of heterogeneity of variances. The percentage of variation in the size of the adenoma between diagnosis and the last assessment was calculated in accordance with the following formula: [(size_diagnosis_ – size_end_)/size_diagnosis_] × 100. The cumulative rate of patients controlled for GH or IGF-1 based on time were estimated using the Kaplan–Meier method. Associations between the different prognostic factors and the control of GH, IGF-1 or both were sought in a survival analysis using the log rank test or, for continuous variables, using the Cox proportional hazards model. The data were analysed using STATA 7·0 software (Stata Corporation, TX).

## Results

### Hormonal efficacy

**Fig. 1:**
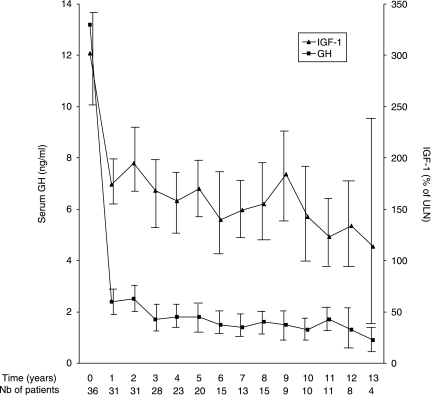
Changes in GH (▪) and IGF-1 (▴) levels in acromegalic patients treated with SSTa (mean ± SE).

Figure 1 shows the decrease in GH and IGF-1 concentrations during primary treatment with SSTa in this cohort of acromegalic patients. The mean (± SEM) pretreatment GH level was 13·5 ± 3·1 µg/l (Table 1), decreasing rapidly at 6 months to 3·8 ± 0·8 µg/l and to 2·4 ± 0·3 µg/l (*P* < 0·01) at 1 year. The GH level decreased slowly over the course of monitoring and was 1·6 ± 0·1 µg/l at 10 years (*P* = 0·02). The mean pretreatment IGF-1 level was 302 ± 26% (expressed as a percentage of the upper limit of the normal range matched for sex and age) (Table 1), decreasing to 181 ± 14% at 6 months, to 174 ± 14% after 1 year (*P* < 0·01), and to 124 ± 18% after 10 years (*P* = 0·02).

**Fig. 2:**
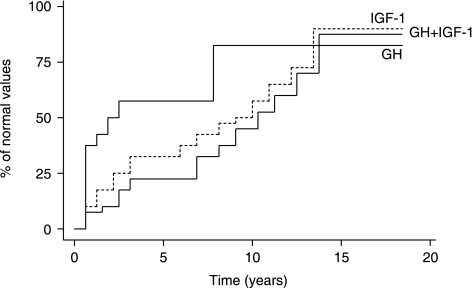
Kaplan–Meier curves: the continuous line represents the level of control of GH. The broken line represents the level of normalization of IGF-1. The bold line represents the control of GH and IGF-1.

Figure 2 shows a progressive increase in the rate of GH control (< 2 µg/l) in a Kaplan–Meier analysis (survival analysis), with 41% controlled at 1 year, 60% at 5 years, and 80% at 10 and 15 years. A progressive increased rate of IGF-1 normalization can be noted, with 17% controlled at 1 year, 31% at 5 years, 57% at 10 years and 87% at 15 years.

In this cohort of acromegalic patients, a GH level < 2 µg/l was obtained in 25 of the 36 patients (70%), a normal IGF-1 level in 24 of the 36 patients (67%), and a GH level < 2 µg/l in combination with a normal IGF-1 level in 21 of the 36 patients (58%). No tachyphylaxis was observed during the long-term SSTa treatment. Furthermore, there was no influence of age or sex on the hormonal efficacy observed during the SSTa treatment.

In hyperprolactinaemic patients treated with SSTa and a dopamine agonist as first-line therapy (*n* = 6), the mean pretreatment GH level was 12·5 ± 2·6 µg/l, decreasing to 3·7 ± 0·9 µg/l at 1 year, and the average pretherapeutic IGF-1 level was 253 ± 35%, decreasing to 212 ± 23% at 1 year.

Concerning the subgroup of patients treated with dopamine agonists as second-line therapy (*n* = 9), there was no further significant decrease in GH or IGF-1 level observed with SSTa alone (Table 2).

**Table 2:** Hormonal and tumour volume changes in the subgroup of patients treated with dopamine agonists as second-line therapy, after 1 year of treatment (*n* = 9)

	GH (µg/l)	IGF-1 (% value over norm)	Tumour volume (mm^3^)
Before treatment	4·1 ± 0·9	271 ± 32	4328 ± 1103
After treatment	5·0 ± 1·3	227 ± 16	3196 ± 1114
*P*	< 0·26	< 0·11	< 0·83

### Tumour control

**Fig. 3:**
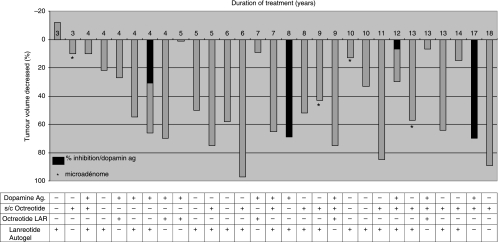
Individual evolution of the tumour volume in acromegalics treated with SSTa. The lined zone represents the ‘gain’ in reduction of volume in patients treated with dopamine agonist. Note that in two patients, the zone is lined in its entirety because the dopamine agonist was introduced at the same time as the SSTa treatment. The asterisk corresponds to microadenomas. The table summarizes the SSTa treatment followed by each patient.

Of the 29 patients analysed, a shrinkage of more than 20% was observed in 21 patients (72%). Figure 3 shows the variation in adenoma volume for each patient. An increase in tumour size was noted in one patient (+13%) without local compressive phenomena while GH/IGF-1 secretion was controlled (mean GH level = 1·49 µg/l, IGF-1 = 98%). The mean reduction in tumour size after 4 years of SSTa treatment was 37%, and 43% (between +13 and –97%) at the last measurement. There was no correlation between the reduction in tumour volume and hormonal control observed during treatment with SSTa. Finally, additional treatment with a dopamine agonist had no benefit in terms of tumour volume reduction (Table 2) in patients primarily treated with SSTa.

### Safety

#### Glucose metabolism

Impaired fasting glucose is defined as a fasting glycaemia > 1·10 g/l. Diabetes mellitus is defined when a fasting glycaemia > 1·26 g/l, according to the criteria of the World Health Organization.

Before any treatment, 30 out of 36 patients did not present with glucose metabolism abnormalities, two patients presented with an impaired fasting glucose, and four patients had diabetes mellitus.

During the long-term treatment with SSTa, the mean glycosylated haemoglobin level remained stable at 6·6 ± 0·3% upon diagnosis, 6·8 ± 0·6% at 5 years, and 6·3 ± 0·36% at 10 years. However, on individual follow-up, four patients developed an impaired glucose tolerance, and four others diabetes mellitus. Conversely, one patient with an impaired glucose tolerance became normoglycaemic.

#### Gall bladder lithiasis

During the course of the study and with systematic evaluation, 13 patients out of 36 (36%) developed sludge and/or gall bladder lithiasis. No patient presented any symptoms in relation to these biliary abnormalities that required treatment to be stopped or a cholecystectomy. The gall bladder lithiasis and/or sludge appeared mainly at the beginning of treatment, within the 4 first years, with the exception of one case of sludge appearing after 10 years of treatment.

#### Pituitary function

At the time of diagnosis, four patients presented with pituitary insufficiency: one isolated gonadotrophin insufficiency (*n* = 1), one gonadotrophin and thyrotrophin insufficiencies (*n* = 1), and two patients with corticotrophin, gonadotrophin and thyrotrophin insufficiencies. No patient presented with a recovery of these pituitary insufficiencies during the SSTa treatment. During the hormonal monitoring, one patient out of the remaining 32 patients developed an isolated thyrotrophin insufficiency (3%).

## Discussion

Treatment of acromegaly aims to keep the secretion of GH and IGF-1 below the thresholds for disease-associated complications, and to reduce the adenoma volume (or limit its expansion) to prevent local compression phenomena and notably optic chiasma. The treatment must be effective and well tolerated to enable satisfactory observance. Finally, the pharmacoeconomic concerns must also be considered.

The aim of this clinical study was to evaluate the long-term efficacy and tolerance of SSTa in a cohort of 36 acromegalic patients responsive to this therapy, when this medical treatment was used as first-line therapy, for an average period of 8 years and with a maximum of 18 years.

In this cohort of acromegalic patients treated with SSTa as first-line therapy, GH control (< 2 µg/l) was obtained in 70% of patients, normal IGF-1 (value adjusted for sex and age) in 67%, and both GH and IGF-1 control in 58%. These results are comparable with the data in the literature reported in Table 3^16–23^ and in recent meta-analyses.^24^,^25^ A rapid and significant reduction in the levels of GH and IGF-1 was noted over the first year of treatment, demonstrating the patients’ response to the SSTa and enabling this therapeutic class to be pursued. Furthermore, there was no tachyphylaxis during the long-term treatment with SSTa of these acromegalic patients. Finally, an increase in GH and IGF-1 control was observed during long-term treatment with SSTa. Several hypotheses can be put forward: a cumulative effect of SSTa concentration over time, an increase in hormonal efficacy of the treatment as reported after the reduction in adenoma volume by surgical debulking,^26^,^27^ although there was no significant correlation between hormonal control and the change in adenoma volume, or a change in the expression of the SST receptor subtypes on the adenoma cells. Nevertheless, the results of the study confirm that SSTa therapy is associated with long-term control of GH/IGF-1 hypersecretion in such patients with acromegaly.

**Table 3:** Comparison of the results observed in our cohort and in the main studies in the literature

Study	Year	Duration of study (months)	No. of patients	% GH control	% IGF-1 control	% GH and IGF-1 control	DEF shrinkage/ % shrinkage (*n*)	Mean decreased volume	Diarrhoea	Lithiasis
Lundin *et al.*^16^	1997	34	18	NA	NA	NA	⇑ Vol > 18/72 (11)	51 (26–67)	NA	NA
Baldelli *et al.*^17^	2000	24	118	77	63	NA	⇑ Vol > 20/22 (23)	NA	46	12
Colao *et al.*^18^	2001	24	36	71	68	NA	⇑ Vol > 25/80 (15)	53 (18–100)	28	8
Ayuk *et al.*^19^	2002	41	22	36	67	NA	NA	NA	NA	23
Bevan *et al.*^20^	2002	6	27	79	53	50	⇑ Vol > 10/96 (27)	49 (12–73)	NA	26
Amato *et al.*^21^	2002	24	20	35	45	NA	⇑ Vol > 10/95 (20)	28	NA	NA
Cozzi *et al.*^22^	2003	30	110	63	65	NA	⇑ Vol > 25/91 (11)	NA	9	18
Cozzi *et al.*^23^	2006	48	67	68·7	70·1	56·7	⇑ Vol > 25/82	62	NA	12
This work	2006	96	36	70	67	58	⇑ Vol > 20/72	43	30	36

NA, not available. The shrinkage was studied in the event of primary treatment (this is why the number of patients is lower than the total number in the various studies).

In some acromegalic patients, the addition of a dopamine agonist did not offer better control of GH/IGF-1 hypersecretion than that obtained during the initial treatment with SSTa. A greater antihormonal efficacy of treatment with dopamine agonists has been observed in the event of a moderate increase in the levels of GH and IGF-1. In such patients, combined dopamine agonist and SSTa treatment offers an additional reduction in the levels of GH and IGF-1 in patients partially responsive to SSTa alone.^28^ It should be noted that *in vitro* studies on GH-adenoma cells of patients partially responsive to SSTa treatment demonstrate that the effect of the SST-DA (BIM23A387) chimeras is mainly linked to the pharmacological effect on the dopamine site.^29^,^30^ Therefore, in patients partially responsive to SSTa used as first-line therapy, several options can be discussed to optimize hormonal control: the addition of a dopamine agonist in case of associated hyperprolactinaemia or when GH/IGF-1 hypersecretion is moderate,^31^ surgical debulking in the case of significant adenoma volume,^26^,^27^ or the addition of pegvisomant (Somavert®) on a weekly basis as was proposed recently.^32^,^33^

Concerning efficacy in relation to tumour size, the mean reduction in adenoma volume was 37% after 4 years and 43% at the last measurement during long-term SSTa treatment, comparable to the results reported in recent meta-analyses,^24^,^34^ and 72% of patients presented an adenoma volume reduction greater than 20%. A moderate increase in tumour mass was noted in one patient with an enclosed macroadenoma, although this increase in size did not lead to a local mass effect, as the adenoma remained separate from the optic chiasma. This patient was controlled on hormonal evaluation. An increase in adenoma volume was reported in 3% of patients on SSTa treatment, while a reduction in tumour volume (between 10% and 25%) was observed in 42% of patients on SSTa therapy in a recent meta-analysis.^34^ The greater efficacy observed in our patients probably relates to the long-term SSTa treatment, and to the fact that the patients had a first-line medical therapy and therefore had not been treated with radiotherapy or surgery, which can lead to periadenomatous fibrosis, reducing the antitumour benefit of the medical treatment.^25^ Furthermore, there was no significant correlation between the reduction in tumour volume and the antihormonal efficacy of SSTa treatment in our study: we observed a significant reduction in GH and IGF-1 levels during the first year whereas a greater antitumour effect is observed after 4 years of treatment in most patients. This dissociation could be linked to different mechanisms of the SSTa in the control of hormonal secretion and tumour reduction.^35^ In particular, the mechanisms of tumour volume reduction during SSTa therapy are still not fully elucidated, and a wide variety of morphological changes have been reported.^36^ Finally, in most GH-secreting pituitary adenoma, regrowth has been reported when SSTa were discontinued, indicating the need for long-term treatment. Thus, long-term treatment with SSTa is associated with tumour growth control in the majority of patients with GH-secreting pituitary adenoma responsive to this treatment.

The tolerance of SSTa treatment is satisfactory and enables its maintenance in acromegalic patients. During primary SSTa treatment no patient stopped the medical therapy due to gastrointestinal, cutaneous or metabolic side-effects. Among the side-effects of transsphenoidal surgery, the risks of rhinorrhoea and meningitis are estimated at 0·8% and 1·8%, respectively, the frequency of secondary pituitary insufficiency is 1·8%, and that of perioperative mortality is 0·1% in the best series.^6^ Complications of surgery have been closely linked to the experience of the neurosurgical team, so a well-trained team is necessary.^5^ Conventional radiotherapy has several disadvantages, including secondary pituitary insufficiency occurring in up to 80% after 10 years;^7^ this complication seems to be less frequent with stereotaxic radiotherapy, but long-term studies are needed to evaluate this treatment.^9^ A significant increase in cerebrovascular mortality,^37^ as well as rare radioinduced cerebral tumours,^38^ have also been reported after conventional radiotherapy. During treatment with SSTa, digestive symptoms such as diarrhoea and/or abdominal pains decrease over time.^39^ During systematic gall bladder ultrasound surveillance, a significant frequency of lithiasis and/or sludge can be noted (36% of cases in our study), in most patients during the first years of treatment, and they were asymptomatic in this series. As suggested previously, it seems unnecessary to recommend systematic ultrasound surveillance during long-term SSTa therapy.^40^ At the metabolic level, mean HbA1C was stable over time, but in eight patients their blood glucose levels deteriorated. Granulomas at the injection site are rare with the use of slow-release forms of SSTa.^39^ Finally, pituitary function was preserved in most cases except for the onset of TSH insufficiency in one patient. Accordingly, long-term SSTa treatment is well tolerated, but this medical treatment requires monitoring of blood glucose levels to adapt antidiabetic treatment, if necessary. However, SSTa treatment is associated with the maintenance of anterior pituitary function in most acromegalic patients.

In conclusion, this clinical study confirms that long-term primary treatment with SSTa is effective on an antihormonal and antitumour level in patients with a GH-secreting pituitary adenoma responsive to SSTa. The treatment is well tolerated and this enables its continued use over the long term. Therefore, primary treatment with SSTa seems to be a valued and validated addition to the therapeutic armamentarium of acromegaly. It seems legitimate to indicate the use of SSTa in the event of contraindication to surgery, refusal by the patient to undergo surgery, normal pituitary MRI or empty sella turcica, and when the success of surgery is unlikely, particularly in case of macroadenoma with large invasion of the cavernous sinus.
